# Phenotypic and Genetic Characterization of the Cheese Ripening Yeast *Geotrichum candidum*

**DOI:** 10.3389/fmicb.2020.00737

**Published:** 2020-05-07

**Authors:** Vincent Perkins, Stéphanie Vignola, Marie-Hélène Lessard, Pier-Luc Plante, Jacques Corbeil, Eric Dugat-Bony, Michel Frenette, Steve Labrie

**Affiliations:** ^1^Department of Food Sciences and Nutrition, STELA Dairy Research Center, Institute of Nutrition and Functional Foods, Université Laval, Quebec City, QC, Canada; ^2^Big Data Research Center, Université Laval, Quebec City, QC, Canada; ^3^Université Paris-Saclay, INRAE, AgroParisTech, UMR SayFood, Thiverval-Grignon, France; ^4^Oral Ecology Research Group, Faculty of Dental Medicine, Université Laval, Quebec City, QC, Canada; ^5^Faculty of Science and Engineering, Department of Biochemistry, Microbiology, and Bioinformatics, Université Laval, Quebec City, QC, Canada

**Keywords:** yeast, cheese, *Geotrichum candidum*, *Galactomyces candidus*, multilocus sequence typing, assimilation of carbon compounds, genome assembly

## Abstract

The yeast *Geotrichum candidum* (teleomorph *Galactomyces candidus*) is inoculated onto mold- and smear-ripened cheeses and plays several roles during cheese ripening. Its ability to metabolize proteins, lipids, and organic acids enables its growth on the cheese surface and promotes the development of organoleptic properties. Recent multilocus sequence typing (MLST) and phylogenetic analyses of *G. candidum* isolates revealed substantial genetic diversity, which may explain its strain-dependant technological capabilities. Here, we aimed to shed light on the phenotypic and genetic diversity among eight *G. candidum* and three *Galactomyces* spp. strains of environmental and dairy origin. Phenotypic tests such as carbon assimilation profiles, the ability to grow at 35°C and morphological traits on agar plates allowed us to discriminate *G. candidum* from *Galactomyces* spp. The genomes of these isolates were sequenced and assembled; whole genome comparison clustered the *G. candidum* strains into three subgroups and provided a reliable reference for MLST scheme optimization. Using the whole genome sequence as a reference, we optimized an MLST scheme using six loci that were proposed in two previous MLST schemes. This new MLST scheme allowed us to identify 15 sequence types (STs) out of 41 strains and revealed three major complexes named GeoA, GeoB, and GeoC. The population structure of these 41 strains was evaluated with STRUCTURE and a NeighborNet analysis of the combined six loci, which revealed recombination events between and within the complexes. These results hint that the allele variation conferring the different STs arose from recombination events. Recombination occurred for the six housekeeping genes studied, but most likely occurred throughout the genome. These recombination events may have induced an adaptive divergence between the wild strains and the cheesemaking strains, as observed for other cheese ripening fungi. Further comparative genomic studies are needed to confirm this phenomenon in *G. candidum*. In conclusion, the draft assembly of 11 *G. candidum*/*Galactomyces* spp. genomes allowed us to optimize a genotyping MLST scheme and, combined with the assessment of their ability to grow under different conditions, provides a reliable tool to cluster and eventually improves the selection of *G. candidum* strains.

## Introduction

The yeast species *Geotrichum candidum* (teleomorph *Galactomyces candidus*) is commonly found in water, air, soil, cereals, ripened fruits, milk, and especially on the surface of mold- and smear-ripened cheeses like Camembert, Tilsit, and Pont-L’Évêque (Marcellino et al., 2001; Boutrou and Guéguen, 2005; Thornton et al., 2010; de Hoog and Smith, 2011; Eliskases-Lechner et al., 2011; Desmasures, 2014). *G. candidum* was long considered an undesirable microorganism because its presence in cheese was uncontrolled, but is now recognized for its contribution to cheese ripening, due to its capacity to consume lactic acid and generate alkaline products (Marcellino et al., 2001; Spinnler et al., 2004; Boutrou and Guéguen, 2005). This yeast also contributes to the development of flavor in the cheese due to its proteolytic and lipolytic activities (Boutrou et al., 2006; Gente et al., 2007). Additionally, *G. candidum* is a good competitor against unwanted microorganisms such as *Mucor* spp. and has an antagonistic action against pathogens such as *Listeria monocytogenes* (Dieuleveux et al., 1998; Boutrou and Guéguen, 2005). However, these various traits, especially those contributing to the development of cheese flavors and texture, are strain dependent. *G. candidum* species display a great diversity at the morphologic and metabolic levels (Guéguen and Schmidt, 1992; Jollivet et al., 1994; Jacobsen and Poulsen, 1995; Molimard et al., 1997). Their high level of polymorphism and widely variable phenotypic traits complicate the identification and characterization of *G. candidum* strains by traditional microbiological methods (Prillinger et al., 1999; Gente et al., 2006). Many indigenous strains of *G. candidum* have been isolated from the environment or from dairy products (Marcellino et al., 2001; Gente et al., 2002b; Lavoie et al., 2012). However, the lack of genomic and functional information has limited their potential utility.

Different *G. candidum* strains can be discerned based on chromosome sizes or sequences of their housekeeping genes (Gente et al., 2002a; Alper et al., 2013; Morel et al., 2015; Jacques et al., 2017). The ability to identify distinct phylogenetic groups within the *G. candidum* species lead to the development of two independent multilocus sequence typing (MLST) schemes to genotype isolates of *G. candidum*, but those were never compared using the same set of strains [MLST2013 (Alper et al., 2013) and MLST2017 (Jacques et al., 2017)]. Moreover, only one genome of the *G. candidum* species has been partially sequenced and subjected to further gene prediction analysis (Morel et al., 2015).

The aim of this study was to combine phenotypic assays and whole genome sequencing to elucidate the phenotypic and genetic diversity of eight *G. candidum* strains that belong to distantly related groups from a previous study using MLST (Alper et al., 2013; Table 1). We also used the newly sequenced genomes to optimize a MLST scheme based on the two MLST schemes available in the literature (Alper et al., 2013; Jacques et al., 2017). The phenotypic and genomic results obtained in this study increase the scientific knowledge on the ripening yeast *G. candidum* and, in the near future, could be used to improve strain selection for cheese ripening processes.

**TABLE 1:** Origins and allelic profiles of the 41 *G. candidum* strains analyzed.

Strain	Geo complex	Lineage	ST	Allele						Origin^a^	References
				*ALA1*	*CDC19*	*SAPT4*	*GLN4*	*PGI1*	*PGM2*		
15	A	3	1	1	1	1	1	1	1	Mold-ripened cheese, Canada	Alper et al., 2011
20	A	2	2	1	2	1	1	2	2	Mold-ripened cheese, Canada	Alper et al., 2011
21	C	1	3	2	3	2	2	2	3	Clotted carrot, Japan	Guéguen and Jacquet, 1982
28	A	5	4	1	1	1	1	1	4	Mold-ripened cheese, Canada	Alper et al., 2011
34	A	2	2	1	2	1	1	2	2	Mold-ripened cheese, Spain	Alper et al., 2011
37	A	2	2	1	2	1	1	2	2	Smear cheese, France	Alper et al., 2011
38	A	2	2	1	2	1	1	2	2	Mold-ripened cheese, France	Alper et al., 2011
39	A	5	4	1	1	1	1	1	4	Smear cheese, France	Alper et al., 2011
**40**	**A**	**5**	**4**	**1**	**1**	**1**	**1**	**1**	**4**	**Smear cheese, France**	Dieuleveux et al., 1997
48	B	4	5	1	1	2	3	2	4	Bioreactor contaminant	Alper et al., 2011
**70**	**C**	**1**	**3**	**2**	**3**	**2**	**2**	**2**	**3**	**Smear cheese, Canada**	Alper et al., 2011
73	A	5	4	1	1	1	1	1	4	Smear cheese, France	Gente et al., 2002a, b, 2006
74	B	4	6	1	4	2	1	3	5	Grass, France	Gente et al., 2002a, b, 2006
75	B	4	7	1	5	2	1	4	4	Corn silage, France	Gente et al., 2002a, b, 2006
76	A	5	8	1	1	1	1	2	4	Milk, France	Gente et al., 2002b, 2006
**77**	**A**	**3**	**1**	**1**	**1**	**1**	**1**	**1**	**1**	**Milk, France**	Gente et al., 2002b, 2006
**244**	**C**	**4**	**9**	**3**	**3**	**2**	**4**	**2**	**6**	**Milk, Canada**	Lavoie et al., 2012
**317**	**B**	**4**	**10**	**4**	**4**	**2**	**3**	**2**	**7**	**Organic milk, Canada**	Lavoie et al., 2012
436	A	2	2	1	2	1	1	2	2	Industrial strain–company A	Alper et al., 2011
562	A	5	4	1	1	1	1	1	4	Milk, MPP 1, Canada	Lavoie et al., 2012
**563**	**A**	**5**	**4**	**1**	**1**	**1**	**1**	**1**	**4**	**Milk, Canada**	Lavoie et al., 2012
645	A	5	4	1	1	1	1	1	4	Smear cheese, Canada	Lavoie et al., 2012
655	A	5	4	1	1	1	1	1	4	Mold-ripened cheese, Canada	Lavoie et al., 2012
664	B	4	11	4	4	2	1	2	4	Milk, MPP 1, Canada	Lavoie et al., 2012
690	B	4	12	5	6	2	1	4	7	Milk, MPP 3, Canada	Lavoie et al., 2012
1024	A	3	13	5	1	1	1	1	1	Industrial strain–company B	Alper et al., 2011
1025	A	3	13	5	1	1	1	1	1	Industrial strain–company B	Alper et al., 2011
1026	A	4	14	1	1	1	1	2	8	Industrial strain–company B	Alper et al., 2011
**1028**	**A**	**2**	**2**	**1**	**2**	**1**	**1**	**2**	**2**	**Industrial strain–company A**	Alper et al., 2013
1031	A	3	13	5	1	1	1	1	1	Industrial strain–company A	Alper et al., 2013
1032	A	3	13	5	1	1	1	1	1	Industrial strain–company A	Alper et al., 2013
1033	A	3	13	5	1	1	1	1	1	Industrial strain–company A	Alper et al., 2013
1034	A	2	2	1	2	1	1	2	2	Industrial strain–company A	Alper et al., 2013
1035	C	1	15	3	4	2	2	2	3	Industrial strain–company A	Alper et al., 2013
1036	A	2	2	1	2	1	1	2	2	Industrial strain–company A	Alper et al., 2013
1037	A	3	13	5	1	1	1	1	1	Industrial strain–company A	Alper et al., 2013
1038	A	5	4	1	1	1	1	1	4	Industrial strain–company A	Alper et al., 2013
1039	A	5	4	1	1	1	1	1	4	Industrial strain–company A	Alper et al., 2013
1040	A	5	4	1	1	1	1	1	4	Industrial strain–company A	Alper et al., 2013
1041	A	3	1	1	1	1	1	1	1	Industrial strain–company A	Alper et al., 2013
**1146**	**A**	**3**	**1**	**1**	**1**	**1**	**1**	**1**	**1**	**Artisanal sheep cheese, Slovakia**	**CBS 11176**

*^a^MPP: Milk Production Plant. Bold: Strains sequenced in this study.*

## Materials and Methods

### Biological Material, Culture Conditions, and Genomic DNA Extraction

Eight *G. candidum* (identified in bold in Table 1) and three *Galactomyces* spp. strains were used in this study (Table 2). The eight *G. candidum* strains were selected as representatives of each branch in the phylogenetic tree obtained with MLST2013 (Alper et al., 2013), while the *Galactomyces* spp. strains were chosen to form an outgroup of other *Galactomyces* species. Considering that the genus *Galactomyces* is not well known, the choice of three strains of different species as outgroups allowed to draw a wider portrait of this genus. All strains were grown on YEG (Yeast Extract Glucose) agar plates [10 g⋅L^–1^ of yeast extract (Thermo Fischer Scientific), 10 g⋅L^–1^ of D-glucose (EMD Chemicals) and 15 g⋅L^–1^ of Bacto agar (BD Diagnostics)]. The strains were inoculated directly from 15% glycerol (*v/v*) stock cultures stored at −80°C. The plates were incubated in the dark for 4 days at 25°C. Isolated colonies were re-streaked on YEG agar plates or in YEG Broth and incubated for 7–9 days at 25°C in the dark. An additional 33 *G. candidum* strains isolated from dairy environment was used to validate the newly designed MLST scheme (Table 1).

**TABLE 2:** Identification and origins of the *Galactomyces* spp. strains.

Species	Strain	Origin	References
*Galactomyces geotrichum*	1147	Soil extract, Puerto Rico	CBS 772.71
*Galactomyces reessii*	1148	Cold water retting, Indonesia	CBS 179.60
*Galactomyces citri-aurantii*	1150	Orange orchard soil extract, California	CBS 175.89

The mycelia on YEG Broth were harvested and grounded into fine powder in liquid nitrogen using a CryoMill apparatus (Retsch, Germany). Cryogenic grinding was performed at −196°C with an automatic pre-cooling step at a 5 Hz frequency followed by a 2 min grinding step at a 25 Hz frequency. Genomic DNA was extracted from 30 mg of grounded mycelium using Purelink RNA/DNA viral mini kit (Invitrogen) with the following modifications. Grounded mycelium was homogenized in 200 μl of a 0.9% NaCl solution, prior to the addition of the proteinase K and the lysis buffer. DNA concentration and quality were measured using a NanoDrop ND-1000 spectrophotometer (Thermo Fisher Scientific Inc., Wilmington, NC, United States). The purified DNA was stored at −80°C until used.

### Isolation of Arthrospores, Phenotype Assessment, Growth at 35°C and Assimilation of Carbon Compounds

The arthrospores of eight *G. candidum* and three *Galactomyces* spp. strains were isolated from the mycelium with a sterile cotton swab using 0.5% (*v/v*) Tween 80, suspended in YNB starvation broth [6.7 g⋅L^–1^ of Bacto Yeast Nitrogen Base without amino acids (BD Diagnostics) and 100 g⋅L^–1^ of D-glucose (EMD Chemicals)], and incubated for 48 h at 25°C in the dark (Kurtzman et al., 2011b). Microscopic images were taken, and spore concentration was determined using a hemocytometer and optical microscopy (BX61, Olympus). Thereafter, the solution was diluted with sterile carbon-free YNB solution to obtain a concentration of 1⋅10^6^ spores⋅ml^–1^. In order to compare the morphology between strains, isolated colonies were re-streaked in three points on YEG agar plates and photographed after 7 days of incubation at 25°C using a standardized photography setup (Pitt and Hocking, 2009).

Their ability to grow at 35°C was evaluated by inoculating *G. candidum/Galactomyces* spp. strains on GYP agar plates [40 g⋅L^–1^ of D-glucose (EMD Chemicals), 5 g⋅L^–1^ of yeast extract (Thermo Fischer Scientific), 5 g⋅L^–1^ of Bacto peptone (BD Diagnostics) and 20 g⋅L^–1^ of Bacto agar (BD Diagnostics)] for 4 days at 35°C in sealed plastic bags (containing O_2_) immersed in a water bath to ensure a constant temperature (Kurtzman et al., 2011b). Plates were inoculated in a single point with 1 μl from the starved and diluted yeast cell suspension (1⋅10^6^ spores⋅ml^–1^). Growth was assessed by the presence or the absence of a colony. Preliminary screening of carbon compound assimilation abilities was performed on the same 11 *G. candidum/Galactomyces* spp. strains using YT MicroPlates (BIOLOG) according to the manufacturer’s protocol, with the following modifications. All wells of the microplate were filled with 100 μl of the starved and diluted yeast cell suspension (1⋅10^6^ spores.ml^–1^). Plates were incubated in the dark for 10 days at 25°C in unsealed plastic bags to avoid drying out of the wells. *Yarrowia lipolytica* LMA-23 was used as a positive control, while the negative control consisted of sterile distilled water. Yeast growth was determined after 3 and 10 days by optic density (OD) measurement with a BioTek microplate reader Synergy HI and Gen5^TM^ software v2.07. A scan area measurement of the OD was done at a wavelength of 590 nm. Carbon compounds, namely adonitol (Acros Organics), D-arabitol (Acros Organics), D-(-)-amygdalin (Acros Organics), bromosuccinic acid (Sigma-Aldrich), α-ketoglutaric acid (Sigma-Aldrich), L-(-)-malic acid (Acros Organics), L-glutamic acid (MP Biomedicals), for which variations in assimilation profiles were observed between strains (data not shown) were selected for further tests. Additional carbon sources, namely lactose (Laboratoire MAT), D-glucono-1,5-lactone (Sigma-Aldrich), sodium citrate (BDH), sodium DL-lactate (Sigma-Aldrich) were also selected because their assimilation allows the formal identification of *Geotrichum* and *Galactomyces* strains at the species level and because of their relevance in the dairy environment (de Hoog and Smith, 2011; Hill and Kethireddipalli, 2013). Each carbon source was added at 0.5% concentration (w/v) in 4.9 ml of YNB culture media. For acidic or basic compounds, the pH of the solutions was adjusted between 5.2 and 5.6, with HCl (1N) or NaOH (1N). Spores of all *G. candidum* and *Galactomyces* spp. strains (Tables 1, 2) were then inoculated as described for the YT MicroPlates protocol, in triplicate, using the standard method of Wickerham and Burton and incubated for 21 days at 25°C in the dark (Wickerham and Burton, 1948; Kurtzman et al., 2011b). A 15 s vortex agitation was done on each test tube at days 7 and 21. Growth evaluation was determined by eyes using the descriptors established by Kurtzman et al. (2011b).

### Library Preparation, DNA Sequencing and Genome Assembly

Sequencing library construction was performed from 50 ng of total genomic DNA using the Illumina Nextera DNA library preparation kit (Illumina, San Diego, CA, United States) and Nextera index kit (Illumina), according to the manufacturer’s instructions. Purified libraries were quantified using PicoGreen (Promega, Madison, WI, United States), diluted at 2 μM, multiplexed and sequenced on the Illumina HiSeq 2000 platform (101 bp paired-end reads). For strain LMA-244, the Illumina MiSeq platform (300 bp paired-end reads) was also used. FASTQ file generation and demultiplexing were performed using bcl2fastq v1.8.4 (Illumina, San Diego, CA, United States). Both single- and paired-end reads generated were quality filtered using FastQc v0.11.4 and trimmed with Trimmomatic v0.35 in order to cut adapters or indexes, to remove low quality bases (quality score <10) from the end of the reads, and to discard reads below 21 nt (Andrews, 2010; Bolger et al., 2014). Reads that remained unpaired were retained as single-end reads. Genome assembly was performed using SPAdes v3.11.1 (Bankevich et al., 2012). The genome of strain CLIB 918 as reference was used for the assembly of the eight *G. candidum* genomes (Morel et al., 2015) and *de novo* sequencing and assembly was performed for the *Galactomyces* strains. Based on the optimization of the total number of contigs, the number of contigs ≥1,000 bp and the N50 value, the k-mer option -k 21, 33, 55, 77 were used for all strains.

Scaffolds were filtered using the khmer software with a length cut-off of 1,000 bp (Crusoe et al., 2015). Genome assembly statistics and completeness were assessed using QUAST web interface and BUSCO v3 (Gurevich et al., 2013; Waterhouse et al., 2017). BUSCO measures the completeness of a genome assembly by comparing predicted genes in the assembly to a dataset of near-universal single-copy orthologs selected from OrthoDB v9 (Waterhouse et al., 2017).

### Genomic Content Comparison

A measure of the similarity between all the strains was generated by comparing the k-mer (31 nt) content (presence or absence) of the whole assembled genomes (scaffolds) using Ray Surveyor software, which is a functionality of Ray v3.0.0 (Deraspe et al., 2017). Considering the set of all 31 bp k-mers of an assembled genome *i*, *A_*i*_* = (*k*_1_, *k*_2_, …, *k_*l*__*A*_*), and the set of all 31 bp k-mers of an assembled genome *j, A*_*j*_ = (*k*_1_, *k*_2_,…, *k_*l*__*B*_*), the set of all 31 bp k-mers shared between the assembled genomes *i* and *j* is defined as *K*_*i,j*_ = (*A*_*i*_ ∩ *A*_*j*_). In order to have comparable values because of the varying genome sizes of the *G. candidum* and *Galactomyces* spp. strains, the normalized count of all 31 bp k-mers shared between the assembled genomes *i* and *j*, defined as $K_{i,j}^{\prime }=\frac{\left|k_{i,j}\right|}{\sqrt{\left|k_{ii}\right|\times \left|k_{jj}\right|}}$, was rather used. Hence, a similarity heatmap was generated with gplots packages in the R environment using the values of the normalized set *K′_*i,j*_* of shared 31 bp k-mers for all pairwise strain combinations (Warnes et al., 2016). These normalized set of values were also transformed in Euclidean distance metrics with the distance matrix computation function in the R environment and used to generate a UPGMA dendrogram with MEGA v6 (Tamura et al., 2013; R Development Core Team, 2015).

### MLST Loci Selection

Two MLST schemes were developed and published independently for *G. candidum* isolates typing. MLST2013 targets six housekeeping genes coding for an alanyl-tRNA synthetase (*ALA1*), a pyruvate kinase (*CDC19*), an acetyl-CoA acetyltransferase (*ERG10*), a glutaminyl-tRNA synthase (*GLN4*), a phosphoglucoisomerase (*PGI1*), and a phosphoglucomutase *(PGM2*) (Alper et al., 2013). MLST2017 targets five loci coding for a nucleoporin (*NUP116*), a dihydroorotate dehydrogenase (*URA1*), an oritidine-5′-phosphate decarboxylase (*URA3*), a yeast subtilisin-like protease III (*SAPT4*), and a phospholipase B (*PLB3*) (Jacques et al., 2017).

All 11 loci targeted in MLST2013 and MLST2017 were amplified for the eight isolates of *G. candidum* (identified in bold in Table 1), using the primers available in Table 3 and the following parameters: initial denaturation step of 2 min at 94°C, followed by 35 cycles (denaturation 30 s at 94°C, annealing for 30 s at 56°C and extension of 1 min at 72°C) and a final extension of 3 min at 72°C. PCR products were visualized by electrophoresis on 0.8% agarose gel, stained with Gel Red, and illuminated under UV light. For all primer sets, a single band was obtained. PCR fragments were sequenced using an ABI 3730×l Data Analyzer (Thermo Fisher Scientific, Waltham, MA, United States) at the Plate-forme de séquençage et de génotypage des génomes (Centre de recherche du CHU de Québec, Université Laval, Québec, Canada).

**TABLE 3:** Primer sequences of 11 MLST target loci.

Locus	Primer sequence 5′→ 3′	Locus size (bp)	References
*ALA1*	Fwd-GCTCTTCGTGAGGTTCTTGG	424	Alper et al., 2013
	Rev-ACCTCGTAGGCATCAGTGCT		
*CDC19*	Fwd-CGCCAGTCAGAGAAGGAATA	355	Alper et al., 2013
	Rev-GTCGACCTGGTTCTTGACAC		
*ERG10*	Fwd-AACACAACATTTCCCGTGAG	652	Alper et al., 2013
	Rev-AGAGCTTAGCGTCAGCACTGA		
*GLN4*	Fwd-TGTTCTCAGAGGGTTTCCTG	558	Alper et al., 2013
	Rev-CCACATCTGAGGATTGTCGT		
*PGI1*	Fwd-ACCGCTGAGACTCTTCGCAA	472	Alper et al., 2013
	Rev-CTCCATGGAAAGCTGCTGGA		
*PGM2*	Fwd-GAACGGTGTCTACGGTCTTG	548	Alper et al., 2013
	Rev-TCAATGTACAGACGGATGGTC		
*NUP116*	For-ACCGCTACAACTGGATTTGG	425	Jacques et al., 2017
	Rev-GAGACCTGTTTGAGGGCTTG		
*URA1*	For-CAAGCCAATTGTGCTGAGAA	465	Jacques et al., 2017
	Rev-GGTGTCGTAGGGCAGTTGAT		
*URA3*	For-GCCAAAAAGACCAACCTGTG	470	Jacques et al., 2017
	Rev-CCTCATCCATACGGTTCTGC		
*SAPT4*	For-ATCATTAACACCCCGGCATA	501	Jacques et al., 2017
	Rev-GTGTCACCAAGCAGAGCAAA		
*PLB3*	For-AAGAATATCTGGGATCTTTC	393	Jacques et al., 2017
	Rev-TGAAGAAGAAGTACCAAGAA		

The dendrogram obtained with Ray Surveyor software was used as a reference to compare the phylogenetics trees constructed with the MLST2013 and MLST2017 schemes. Once the optimum consensus MLST scheme was designed, all six target loci (*ALA1, CDC19, SAPT4, GLN4, PGI1*, and *PGM2*) (Table 3) were amplified, using the same PCR conditions, on an additional set of 33 *G. candidum* strains (Table 1).

### MLST Data Treatment and Bioinformatics Analysis

The Staden Package was used for alignment, edition and construction of consensus sequences based on the ABI sequence chromatograms (Staden, 1996). Consensus sequences were entered in Unipro UGENE v1.31.1 software and multiple sequence alignments were performed using MUSCLE (Edgar, 2004; Okonechnikov et al., 2012). Sequences were trimmed to be in frame and encode the same number of amino acids. Evolutionary histories were inferred using the PhyML Maximum-likelihood method with bootstrap test of 1,000 replicates (Felsenstein, 1985).

For the new consensus MLST scheme (MLST2019), the number of polymorphic sites, the dN/dS ratio (dN is the number of non-synonymous substitutions per non-synonymous site and dS is the number of synonymous substitutions per synonymous site), and the average number of nucleotide differences between populations were determined using DnaSP software v6 (Rozas et al., 2017). The allelic profile of each locus was determined based on nucleotide polymorphisms. The allelic profile of each locus was then assigned a unique number in order of discovery (e.g., *Ala1-1, Ala1-2*…). Afterward, each unique combination of six loci numbers (1-1-1-1-1-1) was used to determine the sequence type (ST; e.g., ST1) of each strain (Jolley and Maiden, 2014).

### Phylogenetic Analysis

The MLST2019 data were used to predict the population structure of *G. candidum* by using the STRUCTURE v2.3.4 software (Pritchard et al., 2000). Analyses were performed with a length of Burnin period of 80,000 and 80,000 rounds of calculation after the Burnin period. The number of assumed populations was determined by running simulations with *K* = 2 up to *K* = 10 with five iterations for each assumed population (Porras-Hurtado et al., 2013), and was best estimated when the log (probability of data) ceased to increase rapidly. The population structure was then calculated with the optimal ancestral subpopulation (*K* = 5) with default parameters, including 30 iterations of the analysis for *K* = 5. Recombination analysis was performed for the whole data set, within and between the Geo complexes using the NeighborNet method and the *phi*-test for recombination implemented in the software SplitsTree v4 (Bryant and Moulton, 2004; Huson and Bryant, 2005). Additionally, the linkage disequilibrium between and within the loci was verified with the Index of Association (*I*_*A*_) and the Standardized Index of Association (*I*_*A*_^*S*^) using the LInkage ANalysis (LIAN) software v3.7 (Smith et al., 1993; Haubold and Hudson, 2000).

### Data Availability

Raw sequence data for the Whole Genome Shotgun projects were deposited at GenBank under the Bioprojects accession numbers PRJNA482576 for LMA-40, PRJNA482605 for LMA-70, PRJNA482610 for LMA-77, PRJNA482613 for LMA-244, PRJNA482616 for LMA-317, PRJNA490507 for LMA-563, PRJNA482619 for LMA-1028, PRJNA490528 for LMA-1146, PRJNA486748 for LMA-1147, PRJNA486749 for LMA-1148, and PRJNA486756 for LMA-1150.

MLST partial gene sequences were deposited at GenBank under the accession numbers from MH745581 to MH745588 for *ALA1*, from MH745589 to MH745596 for *CDC19*, from MH745597 to MH745604 for *ERG10*, from MH745605 to MH745612 for *GLN4*, from MH745613 to MH745620 for *NUP116*, from MH745621 to MH745628 for *PGI1*, from MH745629 to MH745636 for *PGM2*, from MH745637 to MH745644 for *PLB3*, from MH745645 to MH745685 for *SAPT4*, from MH745686 to MH745693 for *URA1*, and from MH745694 to MH745701 for *URA3*.

## Results

### Colony Morphology

**FIGURE 1:**
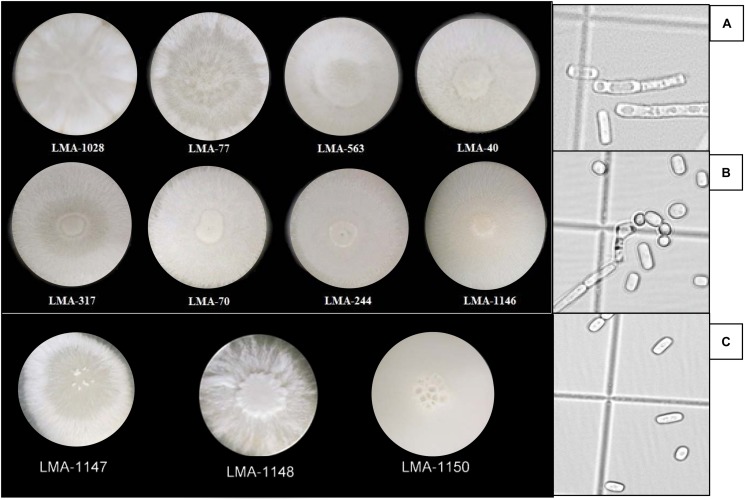
Morphology of *Geotrichum candidum* and *Galactomyces* spp. strains on YEG culture media. (Left) Macroscopic observation of colonies. (Right) Microscopic observation of arthrospores. **(A)** Mold-like (example: LMA-1028). **(B)** Intermediate (example: LMA-244). **(C)** Yeast-like (example: LMA-1146).

Macroscopic and microscopic observations on YEG culture media revealed three different morphotypes among the 11 *G. candidum* and *Galactomyces* spp. strains (i.e., yeast-like, intermediate or mold-like morphotypes) (Figure 1), as previously described (Boutrou and Guéguen, 2005). Strains LMA-77, 563, 1028, and 1148 were characterized by mold-like, white, filamentous, resistant-to-the-touch and not-greasy colonies, with a predominance of vegetative hyphae, whereas LMA-1146 and 1150 were characterized by yeast-like, cream-colored, not resistant-to-the-touch and greasy colonies, with abundant production of arthrospores. Strains LMA-40, 70, 244, 317, and 1147 showed an intermediate morphology, characterized by white-felted, slightly resistant-to-the-touch and not-greasy colonies, with abundant production of arthrospores.

### Carbon Assimilation Profile

**FIGURE 2:**
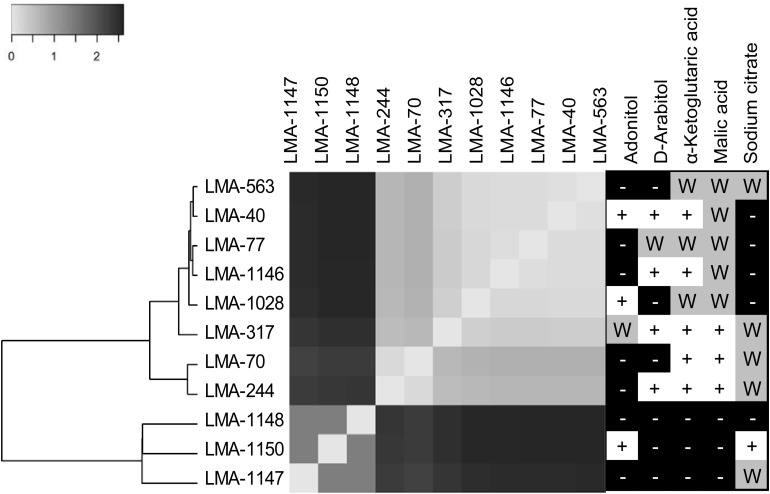
Genomic similarity of three *Galactomyces* spp. and eight *G. candidum* strains represented by a heatmap obtained from the Euclidean distance between the normalized set of k-mers (31 bp fragments) shared between the partial genomes of *Galactomyces* spp. and *G. candidum* strains. Phenetic tree analyses were conducted in MEGA6. (Right panel) Ability of *G. candidum/Galactomyces* spp. strains to grow on carbon compounds (+ for positive assimilation, − for negative assimilation, and W for weak assimilation).

These 11 *G. candidum* and *Galactomyces* spp. strains were previously identified by rDNA sequencing (Alper et al., 2011). To confirm their identification at the species level, we performed carbon assimilation tests in YT MicroPlates and growth tests at 35°C on GYP agar (Table 4). Most of the carbon compounds tested showed similar assimilation profiles for all *G. candidum* and *Galactomyces* spp. strains. A positive assimilation was observed for glucose, galactose, sorbose, xylose, glycerol, and succinate. A negative assimilation was observed for inuline, sucrose, raffinose, melibiose, lactose, trehalose, maltose, methyl-glucoside, cellobiose, salicin, rhamnose, ribose, gluconate, glucosamine, and xylitol. A variable assimilation profile was observed for D-arabitol, α-ketoglutarate, malate, L-arabinose, adonitol, citrate, gluconolactone, lactate, and bromosuccinate. Among these variable assimilation profiles, α-ketoglutarate and malate could be used to distinguish *G. candidum* strains (*G. candidus*) from other *Galactomyces* species. Additionally, all *G. candidum* strains showed variable assimilation profiles for five carbon compounds (adonitol, D-arabitol, α-ketoglutaric acid, malic acid, and sodium citrate) (Figure 2, right panel). Apart from strain LMA-1028, all *G.* candidum strains grew positively at 35°C. On the contrary, none of the three *Galactomyces* spp. strains grew at this temperature (Data not shown).

**TABLE 4:** Carbon assimilation profile for the *G. candidum/Galactomyces* spp. strains.

Genus	*Geotrichum candidum*								*Galactomyces* sp.		
Strain (LMA)	77	1146	1028	40	563	317	70	244	1147	1148	1150
Geo complex	A	A	A	A	A	B	C	C			
ST	ST1	ST1	ST2	ST4	ST4	ST10	ST3	ST9			
**Substrate**											

D-arabitol	W	+	−	+	−	+	−	+	−	−	−
α-ketoglutarate	W	+	W	+	W	+	−	−	−	−	−
Malate	+	W	W	W	W	+	W	+	−	−	−
Glucose	+	+	+	+	+	+	+	+	+	+	+
Inuline	−	−	−	−	−	−	−	−	−	−	−
Sucrose	−	−	−	−	−	−	−	−	−	−	−
Raffinose	−	−	−	−	−	−	−	−	−	−	−
Melibiose	−	−	−	−	−	−	−	−	−	−	−
Galactose	+	+	+	+	+	+	+	+	+	+	+
Lactose	−	−	−	−	−	−	−	−	−	−	−
Trehalose	−	−	−	−	−	−	−	−	−	−	−
Maltose	−	−	−	−	−	−	−	−	−	−	−
Methyl-glucoside	−	−	−	−	−	−	−	−	−	−	−
Cellobiose	−	−	−	−	−	−	−	−	−	−	−
Salicin	−	−	−	−	−	−	−	−	−	−	−
Sorbose	+	+	+	+	+	+	+	+	+	+	+
Rhamnose	−	−	−	−	−	−	−	−	−	−	−
Xylose	+	+	+	+	+	+	+	+	+	+	+
L-arabinose	−	−	−	−	−	−	−	−	−	−	W
Ribose	−	−	−	−	−	−	−	−	−	−	−
Glycerol	+	+	+	+	+	+	+	+	+	+	+
Adonitol	−	−	+	+	−	W	−	−	−	−	+
Mannitol	+	+	+	+	+	+	+	+	+	−	+
Succinate	+	+	+	+	+	+	+	+	+	+	+
Citrate	−	−	−	−	W	W	W	W	W	−	+
Gluconate	−	−	−	−	−	−	−	−	−	−	−
Glucosamine	−	−	−	−	−	−	−	−	−	−	−
Xylitol	−	−	−	−	−	−	−	−	−	−	−
Gluconolactone	+	+	+	+	+	+	+	+	+	−	W
Lactate	+	+	+	+	+	+	+	+	W	+	+
Bromosuccinate	+	+	−	+	+	+	+	+	N/A	N/A	N/A
Amygdalin	W	W	W	W	W	W	W	W	N/A	N/A	N/A
Glutamate	+	+	+	+	+	+	+	+	N/A	N/A	N/A

*+: Positive assimilation; −: Negative assimilation; W: Weak assimilation; N/A: Not available.*

### Sequencing, Genome Assembly and Genomic Content Comparison

The statistics for the eight *G. candidum* genome assemblies (Table 1 in bold) were highly variable, except for the assembly size, the GC content and the BUSCO completeness (Table 5). Genome sizes varied from 23.1 Mb (LMA-40) to 24.2 Mb (LMA-70), with an average GC content of 42%. Similarly, the statistics for the three *Galactomyces* spp. *de novo* assemblies were highly variable depending on the species, except for the BUSCO completeness. The genome length varied between 22 Mb (*Galactomyces geotrichum* LMA-1147) and 26 Mb (*Galactomyces citri-aurantii* LMA-1150), and the GC content between 37% (*Galactomyces reessii* LMA-1148) and 40% (*G. geotrichum* LMA-1147). Although different strains and/or species were assembled, for all partial genomes, the BUSCO completeness percentage was close to 90% which suggests a good quality draft genome for all isolates sequenced.

**TABLE 5:** Assembly statistics for the genome of *Geotrichum candidum* and *Galactomyces* spp. strains.

LMA strain	Mean coverage	Assembly size (Mb)	Total no. of scaffolds	Scaffold N50 (bp)	Longest scaffold (bp)	GC content (%)	BUSCO Completeness (%)
40	63×	23.10	1,268	32,639	167,475	41.8	90.3
70	70×	24.17	1,450	44,909	195,322	41.4	90.2
77	58×	23.32	1,488	29,977	158,856	41.7	90.5
244	114×	23.20	836	137,000	210,021	41.5	90.3
317	19×	23.36	1,370	35,529	209,353	41.8	90.3
563	49×	23.30	1,427	31,326	124,845	41.7	89.9
1028	34×	23.42	1,332	36,124	191,607	41.7	90.5
1146	54×	23.35	1,363	32,892	152,162	41.7	90.3
1147	64×	21.96	3,121	13,090	102,582	40.4	89.8
1148	105×	22.56	2,775	13,420	64,496	36.6	89.5
1150	55×	25.98	2,668	16,289	69,389	39	90.9

The similarity among the partially assembled genomes of the *G. candidum* and *Galactomyces* spp. strains was assessed by comparing their k-mer content, using the software implementation Ray Surveyor (Deraspe et al., 2017). The dendrogram generated from the *K*′*_*i,j*_* Euclidean distances and the heatmap representation of the normalized similarity values (*K*′*_*i,j*__)_* uncovered separate groups within the strains (Figure 2, left and center). First, the *G. candidum* strains (LMA-40, LMA-70, LMA-244, LMA-317, LMA-563, LMA-1028, and LMA-1146) were clearly separated from the other *Galactomyces* (strains LMA-1147, LMA-1148, and LMA-1150). These two groups shared 3% or less of their k-mer content. Even though *G. candidum* strains were closely related, they could be separated into three major groups based on their k-mer content (Figure 2).

### Multilocus Sequence Typing Schemes for *G. candidum*

We observed similarities between both previous MLST schemes, according to the clustering of the strains (Supplementary Figures S1–S3). LMA-40, -77, -563, -1028, and -1146 were grouped together whereas LMA-70 and -244 were grouped together at the opposite side of the tree. On the other hand, LMA-317 clustered differently in the MLST2017 scheme compared to MLST2013. In MLST2017, LMA-317 clustered in a third group of isolates located between the other complexes. Also, for LMA-40, -77, -563, -1028, and -1146, clustering between strains of the same clade was different in the two schemes. This observation led us to evaluate the resolution level of both schemes (Supplementary Figures S1, S2) by constructing a maximum-likelihood phylogenetic tree for each scheme. We compared both trees to the dendrogram that we constructed using the distance matrix based on whole genome content (Figure 2 left panel). Both MLSTs showed similar clustering to the one using whole genomic content, but none had the same precision as the whole genome comparison. Following these observations, we selected six (6) target loci from both MLST schemes to develop an improved genotyping method for *G. candidum* isolates (Supplementary Figure S3), named MLST2019. We selected the loci *ALA1, CDC19, SAPT4, GLN4, PGI1*, and *PGM2* because they clustered the isolates the same way as the shared k-mer comparison using the whole genomic content. We then genotyped 33 additional *G. candidum* strains using MLST2019.

**FIGURE 3:**
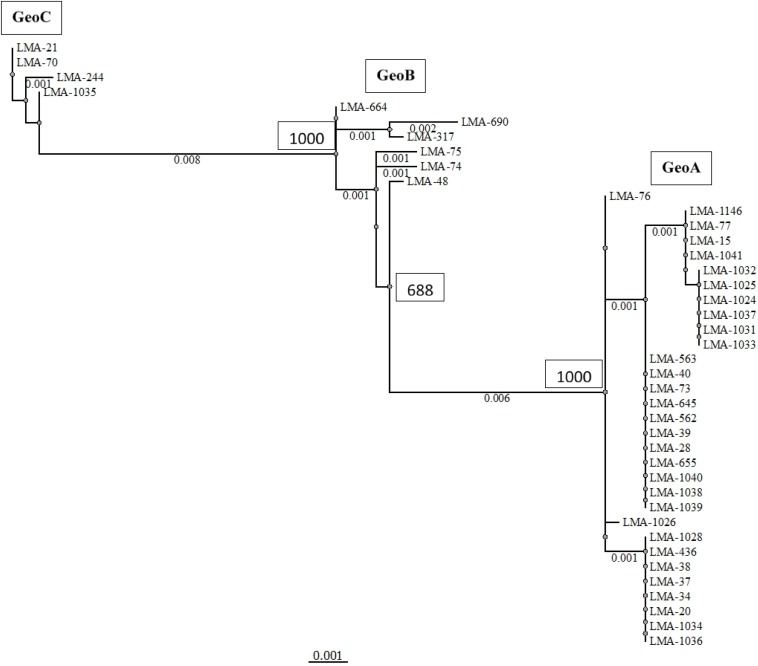
Phylogram of the MLST2019 scheme (*ALA1, CDC19, SAPT4, GLN4, PGI1*, and *PGM2*) constructed with the PhyML Maximum Likelihood method with 1,000 bootstraps (bootstrap values are shown next to the main nodes) on 41 isolates of *Geotrichum candidum*. Phylogenetic analyses were performed with Unipro UGENE v1.31.1.

We applied the MLST2019 scheme to a total of 41 strains of *G. candidum* (for which 40/41 were the same as MLST2013) and observed 15 STs distributed into three complexes, namely GeoA, GeoB, and GeoC (Figure 3). GeoA complex, supported by a bootstrap value of 1, contained 31 isolates, whereas GeoB complex, supported by a bootstrap value of 0.688, contained 6 isolates and GeoC complex, supported by a bootstrap value of 1, contained 4 isolates. Most *G. candidum* strains (31/41) clustered within GeoA, which includes all the commercial starters except LMA-1035 and all the cheese isolates except LMA-70. Furthermore, the GeoA isolates had either the filamentous, intermediate or yeast-like morphotype. The GeoB complex was composed of *G. candidum* isolates with an intermediate or yeast-like morphotype and these were divided into two sub-complexes, according to their isolation source. Dairy strains LMA-317, 664, and 690 isolated from milk produced in Canada clustered separately from the non-dairy strains LMA-48, -74, and -75, isolated respectively from a bioreactor, grass and corn silage (Table 1). The GeoC complex was composed of strains isolated from various environments and were not clustered according to their isolation source. These strains had either a filamentous (LMA-21) or an intermediate (LMA-70, -244, and -1035) morphotype on YEG agar. LMA-1035 was a commercial ripening starter, and LMA-21, -70, and -244 were respectively isolated from rotting carrots, smear cheese and milk (Table 1).

The combined sequences of the six loci used in the MLST2019 scheme generated a concatenated contig of 2,859 bp with 64 polymorphic sites. The number of polymorphic sites varied from 5 for *CDC19* to 18 for *GLN4* and consisted mostly of synonymous substitutions. The number of non-synonymous substitution sites varied between 1 and 2 for most loci and up to 4 sites for the *PGI1* locus. The allelic profile for each locus was 2 for *SAPT4*, 4 for *GLN4* and *PGI1*, 5 for *ALA1*, 6 for *CDC19*, and 8 for *PGM2*. The proportion of variable sites varied between 1.27 and 3.23%. For each locus, the number of alleles was always lower than the number of polymorphic sites, which resulted in a number of variable alleles per variable sites ranging from 0.13 to 1.20. The dN/dS ratio was always close to 0 and mostly lower than 0.2, except for *PGI1* (Table 6).

**TABLE 6:** Loci characteristics targeted in the consensus MLST scheme used to genotype 41 isolates of *G. candidum*.

Locus	Locus size (bp)	Number of polymorphic sites*	Number of alleles	Proportion of variable sites (%)	Number of alleles per variable site	dN/dS
*ALA1*	424	8 (1)	5	1.89	0.63	0.000
*CDC19*	355	5 (1)	6	1.41	1.20	0.171
*SAPT4*	501	16 (2)	2	3.19	0.13	0.079
*GLN4*	558	18 (2)	4	3.23	0.23	0.028
*PGI1*	472	6 (4)	4	1.27	0.67	0.616
*PGM2*	548	11 (1)	8	2.01	0.73	0.048

*Numbers in parentheses are the number of non-synonymous substitution sites detected in the locus.

### Evaluation of the Population Structure of *G. candidum*

**FIGURE 4:**
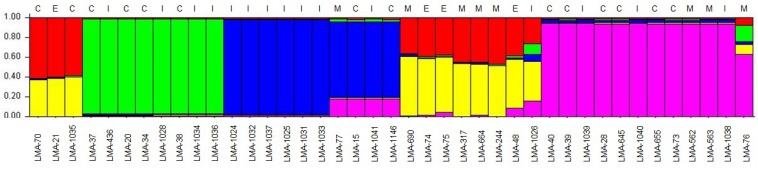
Estimation of the population structure of 41 *G. candidum* strains based on their sequence type profile. For each of the strains, on the abscissa, the estimated contribution, on the ordinate, to its genotype from each of five hypothetical ancestral genotypes is represented by bar plot of different colors. The source of isolation of each strain is shown above the histogram: cheese strain (C), environmental strain (E), industrial strain (I), milk (M). Each color represents a lineage in the *G. candidum* population. Lineage 1 (60% red and 40% yellow), lineage 2 (green), lineage 3 (blue), lineage 4 (close to 60% yellow and 40% red), and lineage 5 (purple).

Using MLST2019 ST profiles in STRUCTURE software, we statistically estimated the number of ancestral genotypes (*K*) of the yeast *G. candidum*. The STs were clustered in 5 subpopulations (*K* = 5), called lineages or clades (1, 2, 3, 4, and 5) (Figure 4 and Table 1). When compared to MLST2019 clusters (GeoA, B, and C), lineages 2, 3, and 5 form the GeoA complex while lineage 1 corresponds to GeoC. The lineage 4 corresponds mostly to GeoB except for strains LMA-1026 and LMA-244, which come from GeoA and GeoC, respectively (Figure 3 and Table 1). The subpopulations observed did not seem to cluster the strains either by their ability to assimilate carbon sources, their colonial morphology or their isolation source, although lineages 2, 3, and 5 do not contain environmental isolates (Table 1 and Figure 4).

**FIGURE 5:**
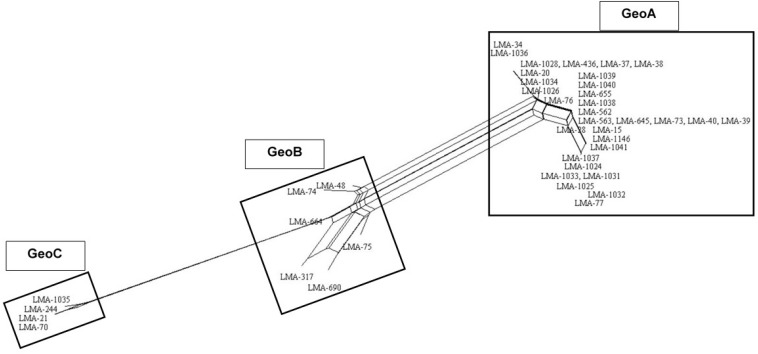
NeighborNet analysis of the combined six loci (*ALA1, CDC19, SAPT4, GLN4, PGI1*, and *PGM2*) obtained from 41 *G. candidum* isolates. Numbering in the figure corresponds to sequence type. Parallelogram formation indicate recombination events. NeighborNet analysis was performed with SplitsTree version 4.

All MLST2019 loci and Geo complexes were investigated for genetic exchanges by a NeighborNet analysis, based on nucleotide sequences. The six targeted loci (*ALA1, CDC19, SAPT4, GLN4, PGI1*, and *PGM2*) showed a tree-like subpopulation structure, which suggests that the allele diversity is due to clonal descent within a given locus rather than recombination (Supplementary Figure S4). The NeighborNet analysis was then performed for GeoA, GeoB, and GeoC complexes separately, for all pairs of complexes GeoA-B, GeoB–C, and GeoA–C, and for all complexes together GeoA–B–C (Figure 5). Tree-like structures were observed within GeoA, and GeoC complexes, and between GeoA and GeoC. The GeoB individual complex and all other comparisons (GeoA–B, GeoB–C, and GeoA–B–C) showed parallelogram-shaped (network-like) structures, suggesting intergenic recombination (data not shown). The recombination hypothesis was verified doing a *phi*-test in SplitsTree (Table 7; Bruen et al., 2006). The recombination events were supported statistically within the GeoB complex (*p* = 0.03), between the GeoA and the GeoB complexes (*p* ≤ 0.001) and on the whole data set (*p* ≤ 0.001) while the absence of recombination was statistically supported for the GeoA (*p* = 1.0), between GeoA and GeoC (*p* = 0.2) and between the GeoB and GeoC (*p* = 0.3). Because only four strains formed the GeoC complex, not enough data was available to perform the *phi*-test.

**TABLE 7:** Recombination and linkage disequilibrium statistics.

Geo group	*Phi*	I_*A*_	I_*A*_^*S*^	*p*-value
A	1.0	0.21	0.4	0.5
B	0.03	–0.33	–0.07	0.9
C	NA	–0.63	–0.13	1
A–B	≤0.001	0.32	0.06	0.05
B–C	0.3	–0.2	–0.04	0.8
A–C	0.2	1.9	0.37	0.0001
A–B–C	≤0.001	0.36	0.07	0.03

Finally, we verified the linkage disequilibrium of all loci within and between the Geo complexes by using the value of the Index of Association (*I*_*A*_) and the Standardized Index of Association (*I*_*A*_^*S*^) calculated using LIAN software. Both indexes measure the extent of linkage equilibrium within a population by quantifying the amount of intergenic recombination and by detecting association among alleles at different loci (Agapow and Burt, 2001). If the value of the indexes differs significantly from zero, it is a sign of linkage disequilibrium and of a clonal population, while a value of 0 is a sign of linkage equilibrium due to frequent recombination (Xu et al., 2014). The *I*_*A*_*^*S*^* values were close to 0 for GeoB, GeoA–B, GeoB–C, and GeoA–B–C complexes, and were significantly different from 0 for GeoA (0.4, *p* = 0.5), GeoC (−0.13, *p* = 1), and GeoA–C (0.37, *p* ≤ 0.0001) complexes, confirming what was observed in the NeighborNet analysis (Table 7).

## Discussion

### Phenotypic Identification and Characterization

The strains used in this study were successfully associated to *G. candidum* or a *Galactomyces* species following their phenotypic characterization (de Hoog and Smith, 2011). The strains LMA-1147 (*G. geotrichum*), LMA-1148 (*G. reessii*), and LMA-1150 (*G. citri-aurantii*) had the typical carbon assimilation profiles and the ability to grow at 35°C. Based on the same criteria, all other isolates except LMA-1028 were positively identified as *G. candidum*. It was also possible to distinguish *G. candidum* strains from the *Galactomyces* species, based on their weak or positive utilization of α-ketoglutaric acid and malic acid (Table 4), as already observed (Kurtzman et al., 2011a). In addition, the pairwise comparison of the shared k-mer content (31-mers) confirmed the identification and phylogeny of the four species studied (Figure 2).

The *G. candidum* strains displayed variability in the assimilation of a few carbon compounds (Figure 2, right panel) which could reflect subgroups with different functional traits. In contrast with previous reports, we found that LMA-40 can use adonitol and did not detect variable assimilation of DL-lactate and D-mannitol among the *G. candidum* strains (Marcellino et al., 2001; Alper et al., 2013). In addition, using traditional microbiological tests, we observed for the first time the variable assimilation of malic acid and α-ketoglutaric acid, the weak assimilation of amygdalin, and the positive assimilation of bromosuccinic acid in *G. candidum*. Some of the carbon compounds tested in this study can be metabolized during cheese ripening by different microorganisms, including *G. candidum* strains. Lactate assimilation by *G. candidum* during the early phase of cheese ripening has been described several times and is positively associated with deacidification of the cheese surface, which is mandatory for the growth of acid-sensitive bacteria (Fox and Law, 1991; Marcellino et al., 2001; Gripon, 2002; Leclercq-Perlat et al., 2004; McSweeney, 2004; Lessard et al., 2014; Arfi et al., 2005; Boutrou and Guéguen, 2005; Castellote et al., 2015; Dugat-Bony et al., 2015). Citrate metabolism in lactic acid bacteria leads to the production of CO_2_ and diacetyl, which contributes to the typical aspect and flavor of Gouda, Cottage, Quark, and Cheddar cheeses (Fox and Law, 1991; Molimard and Spinnler, 1996; McSweeney, 2004). However, citrate utilization as the sole carbon source by *G. candidum* strains was found to be weak or negative (Figure 2, right panel), suggesting that metabolites produced by *G. candidum* through citrate catabolism would not be major contributors to the typical flavor of surface-ripened cheese varieties (Marcellino et al., 2001; Leclercq-Perlat et al., 2004; Lessard et al., 2014). Glutamic acid, which can be consumed by all *G. candidum* strains tested, is produced through the transamination of L-methionine. This enzymatic reaction, in the presence of an amino-acid acceptor, usually the α-ketoglutaric acid, contributes to ammonia and aroma production by *G. candidum* strains during cheese ripening (Brennan et al., 2004; Boutrou and Guéguen, 2005; Cholet et al., 2007; Lessard et al., 2014; McSweeney, 2004). Finally, no relationship was observed between the genetic population structure and the phenotypic characteristics.

### Genome Comparisons and MLST Analysis

Before this study, the only genome sequence of *G. candidum* available in public databases was for the strain CLIB 918, which was isolated from Pont-L’Evêque cheese (Morel et al., 2015). The draft genome assemblies for eight additional *G. candidum* strains presented in this study contained from 836 to 1,488 scaffolds (≥1,000 bp) with N50 values between 29,977 and 137,000 bp (Table 4). The high number of short scaffolds observed might arise from the short reads produced through the Illumina HiSeq technology and the presence of repeated regions, which are abundant in eukaryotic genomes (Paszkiewicz and Studholme, 2010; Yandell and Ence, 2012; Kuo et al., 2014). Using the *G. candidum* CLIB 918 draft genome sequence as a reference allowed major improvements in the scaffolding step for the *G. candidum* strains. The assembly size, N50 value and length of the longest scaffold increased with CLIB 918 as a reference, in comparison to assemblies without CLIB 918 (Supplementary Table S1). Overall, the three *Galactomyces* spp. draft genome assemblies provided similar estimations of the genome sizes and GC contents to those previously proposed using the thermal denaturation method (Smith et al., 1995). These assemblies contain close to 3,000 scaffolds (≥1,000 bp) with N50 value around 13,000 bp. The absence of an available reference genome for the scaffolding of *Galactomyces* spp. strains could explain the more fragmented assemblies, compared to those of *G. candidum* strains.

Sequencing the whole genome of eight *G. candidum* and three *Galactomyces* spp. strains allowed us to use the pairwise comparison of their shared k-mer content as the reference clustering method for optimizing current MLST schemes. Two previous MLST schemes were compared for the genotyping of a common set of eight *G. candidum* isolates (Alper et al., 2013; Jacques et al., 2017). Genotyping with MLST2013 gave rise to two groups of isolates, as previously obtained, whereas a third group was obtained with MLST2017 (Alper et al., 2013; Jacques et al., 2017). Although a few subgroups differ between MLST2013 and MLST2017, both schemes clustered LMA-70 and LMA-244 together and apart from the other *G. candidum* strains.

Considering that neither MLST2013 nor MLST2017 allowed the same sub-grouping as the shared k-mer content, a selection of six loci was used to create an optimized MLST scheme (MLST2019) that would increase the discrimination power between the isolates. The MLST2019 scheme uses the loci *ALA1*, *CDC19, SAPT4, GLN4, PGI1*, and *PGM2*, which, once concatenated, generate a sequence of 2,859 bp. The evolution rate was assessed using the dN/dS ratio for each locus (between 0.000 and 0.616) and suggests that they are not subjected to positive selection, making them suitable gene targets for MLST (Shabbir et al., 2013; Yu et al., 2015).

The evidence for genetic diversity could indicate an adaptive divergence, a population differentiation under selection, between the wild strains and the cheese ripening strains of *G. candidum*, as observed for other fungi used in the agri-food industry such as *Penicillium roqueforti* (Dumas et al., 2018), *Penicillium camemberti* (Cheeseman et al., 2014), and *Saccharomyces cerevisiae* (Legras et al., 2018). This hypothesis is supported by the observation that most of the non-dairy strains are clustered in GeoB and GeoC complexes, whereas GeoA complex contains all industrial cheesemaking strains studied here, except for LMA-1035, and other isolates from milk or cheese. When analyzing the population structure of the *G. candidum* isolates, we observed that the Geo complexes (Figures 3, 5) were similar, but there was a discordance in comparison to the estimation of the ancestral subpopulations (lineages) (Figure 4). The five *G. candidum* lineages were clustered accordingly to MLST2019, except for linage 4 which contains all GeoB strains and includes LMA-244 and LMA-1026. According to the concatenated MLST loci and the whole genome sequences, these strains are part of GeoC and GeoA complexes, respectively (Figures 3, 5). The fact that LMA-244 and -1026 share alleles with strains of the complex GeoB could hint at a speciation of environmental strains (GeoB) to form new complexes GeoA and GeoC (Dumas et al., 2018). Additionally, the calculation of *I*_*A*_ and *I_*A*_^*S*^* indices, both close to 0, supports the hypothesis that the strains studied are in linkage equilibrium and that recombination did play a role in increasing the STs diversity, which was statistically demonstrated, except for GeoB–C (Table 7; Jolley et al., 2001; Cangi et al., 2016). In this particular case, the low number of strains (LMA-21, -70, -244, and -1035) in the GeoC complex might explain the discordance between both analysis. The isolation and genome sequencing of more strains clustering in GeoC would be needed to further characterize the species *G. candidum*. Moreover, a lower diversity was observed in the GeoA complex than in GeoB and GeoC, as represented by the fewer number of branches and their smaller size (Figure 3). A similar phenomenon has also been observed for domesticated *P. roqueforti* strains, for which cheese complexes had fewer and smaller branches compared to the other clusters (Dumas et al., 2018). Furthermore, GeoC isolates were distant from the GeoA/B complexes on a phylogenetic level, and isolates from the environment (LMA-21) and the dairy industries (LMA-70, -244, and -1235) were obtained for the GeoC complex. These findings suggest that the GeoC complex could represent a new pool of *G. candidum* strains with an interesting biotechnological potential for the development of cheese products with typical properties.

## Conclusion

In conclusion, the aim of this study was to combine traditional microbiological techniques with new whole genome shotgun sequencing to illuminate the phylogenetic relationships among *G. candidum* and *Galactomyces* spp. strains of environmental and dairy origin. Thereby, we report the draft assembly of 11 *Geotrichum*/*Galactomyces* spp. strains, increasing the amount of genomic data available for *G. candidum* and other *Galactomyces* species, for which no genome sequence assemblies were available. These strains were identified based on their ability to grow under different conditions and we propose a new MLST scheme to optimally genotype *G. candidum* isolates, because it better represents the whole genomic content of this species and allows a clear visualization of its population structure. Further studies are needed to test the adaptive divergence hypothesis and future comparative genomics studies will shed light on the differences between GeoC and GeoA/B. Also, the sequencing of additional genome, especially from non-dairy niche, are likely to improve the new MLST scheme and give a better understanding of the diversity within each complex. Phenotypic and genomic tools such as whole genome sequencing and the MLST scheme substantially improve our knowledge on this ripening yeast and could ultimately allow a better selection and control of *G. candidum* strains throughout the ripening of cheeses.

## Data Availability Statement

The datasets generated for this study can be found in the raw sequence data for the Whole Genome Shotgun projects were deposited at GenBank under the Bioprojects accession numbers PRJNA482576 for LMA-40, PRJNA482605 for LMA-70, PRJNA482610 for LMA-77, PRJNA482613 for LMA-244, PRJNA482616 for LMA-317, PRJNA490507 for LMA-563, PRJNA482619 for LMA-1028, PRJNA490528 for LMA-1146, PRJNA486748 for LMA-1147, PRJNA486749 for LMA-1148, and PRJNA486756 for LMA-1150. MLST partial gene sequences were deposited at GenBank under the accession numbers from MH745581 to MH745588 for ALA1, from MH745589 to MH745596 for CDC19, from MH745597 to MH745604 for ERG10, from MH745605 to MH745612 for GLN4, from MH745613 to MH745620 for NUP116, from MH745621 to MH745628 for PGI1, from MH745629 to MH745636 for PGM2, from MH745637 to MH745644 for PLB3, from MH745645 to MH745685 for SAPT4, from MH745686 to MH745693 for URA1, and from MH745694 to MH745701 for URA3.

## Author Contributions

VP, SV, M-HL, ED-B, MF, and SL were involved in planning the study and writing the manuscript. SV performed phenotypic characterization, DNA extraction, and genome assembly and comparative genomics analysis. M-HL performed DNA libraries for HiSeq sequencing. VP performed DNA extraction for MLST schemes comparison, MLST analysis, and genome assembly and comparative genomics analysis. P-LP and JC helped with the development of the bioinformatic scripts used for genome analysis.

## Conflict of Interest

The authors declare that the research was conducted in the absence of any commercial or financial relationships that could be construed as a potential conflict of interest.
