# Supplementary material for: Phenotypic and Genetic Characterization of the Cheese Ripening Yeast Geotrichum candidum
Source: Front Microbiol. 2020 May 7;11:737. doi: 10.3389/fmicb.2020.00737 (PMC7220993; doi:10.3389/fmicb.2020.00737)
Supplement: Supplementary file 1 [file Data_Sheet_1.pdf]

*Supplementary Material*

# Phenotypic and Genetic Characterization of the Cheese Ripening Yeast *Geotrichum candidum*

**Vincent Perkins[1], Stéphanie Vignola[1] Marie-Hélène Lessard[1], Pier-Luc Plante[2], Jacques Corbeil[2], Eric Dugat-Bony[1,3], Michel Frenette[4], Steve Labrie[1*]**

[1] Department of Food Sciences and Nutrition, STELA Dairy Research Center, Institute of Nutrition and Functional Foods, Université Laval, Quebec City, QC, Canada,

[2] Big Data Research Center, Université Laval, Quebec City, QC, Canada,

[3] Université Paris-Saclay, INRAE, AgroParisTech, UMR SayFood, Thiverval-Grignon, France.

[4] Oral Ecology Research Group, Faculty of Dental Medicine, Université Laval, Quebec City, QC, Canada,

[5] Faculty of Science and Engineering, Department of Biochemistry, Microbiology, and Bioinformatics, Université Laval, Quebec City, QC, Canada

***Correspondence:**
Steve Labrie
**Steve.Labrie@fsaa.ulaval.ca**

**Supplementary Table S1**. Statistics of the *Geotrichum candidum* genome assembly with and without using the *G. candidum* CLIB 918 genome as a reference

| Strain | LMA-40 | | LMA-70 | | LMA-77 | | LMA-244 | | LMA-317 | | LMA-563 | | LMA-1028 | | LMA-1146 | |
|---|---|---|---|---|---|---|---|---|---|---|---|---|---|---|---|---|
| CLIB 918 reference | Yes | No | Yes | No | Yes | No | Yes | No | Yes | No | Yes | No | Yes | No | Yes | No |
| Assembly size (Mb) | 23.10 | 22.9 | 24.17 | 23.08 | 23.32 | 22.84 | 23.2 | 23.15 | 23.36 | 21.65 | 23.3 | 22.88 | 23.42 | 22.91 | 23.35 | 22.86 |
| Total no. Of scaffolds | 4359 | 5337 | 6182 | 4566 | 7450 | 8374 | 4124 | 4454 | 4432 | 8587 | 6333 | 5818 | 6488 | 7329 | 5331 | 5395 |
| Total no. Of scaffolds >1000 bp | 1268 | 2041 | 1450 | 1495 | 1488 | 2424 | 836 | 1211 | 1370 | 4626 | 1427 | 2313 | 1332 | 2279 | 1363 | 1991 |
| Scaffold N50 (kbp) | 33 | 19 | 45 | 27 | 30 | 16 | 137 | 216 | 36 | 6 | 31 | 17 | 36 | 17 | 33 | 19 |
| Longest scaffold (kb) | 167 | 90 | 195 | 114 | 159 | 103 | 210 | 154 | 209 | 42 | 125 | 91 | 192 | 103 | 152 | 88 |
| GC content (%) | 41.8 | 41.8 | 41.4 | 41.6 | 41.7 | 41.8 | 41.5 | 41.5 | 41.8 | 42.3 | 41.7 | 41.9 | 41.7 | 41.8 | 41.7 | 41.9 |

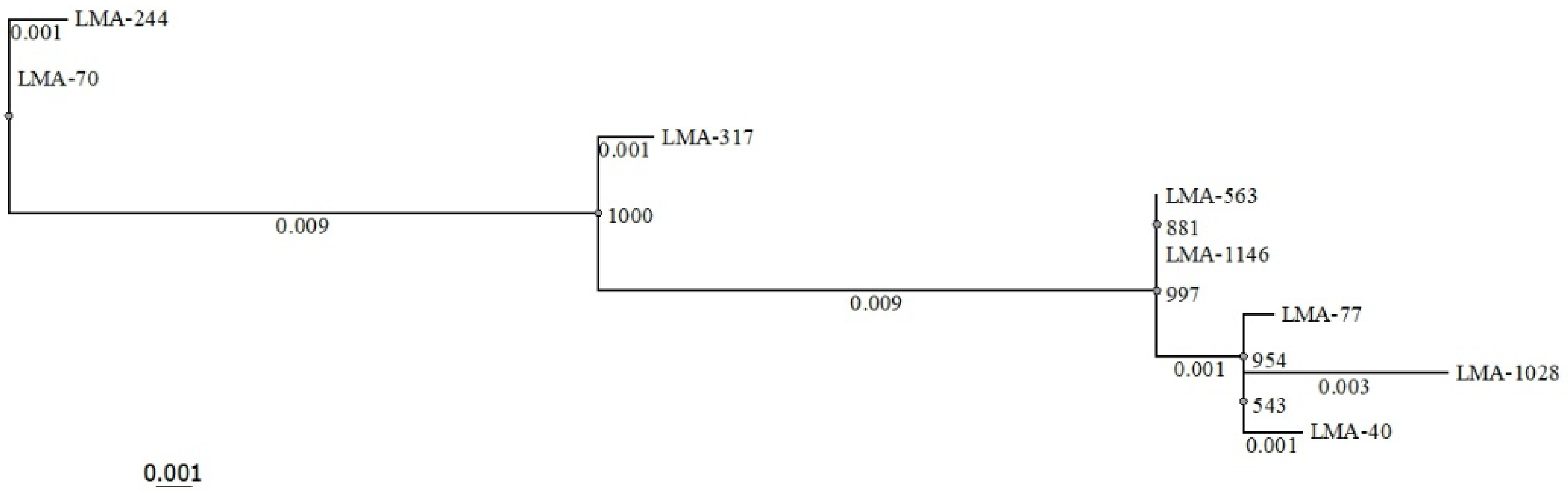


**Figure S1**. Phylograms constructed with the PhyML Maximum-Likelihood method with 1000 bootstraps with 8 isolates of *Geotrichum candidum* for the MLST2017 scheme (*NUP116, SAPT4, URA1, URA3, PLB3*). Phylogenetic analyses were conducted in UGENE v1.31.1.

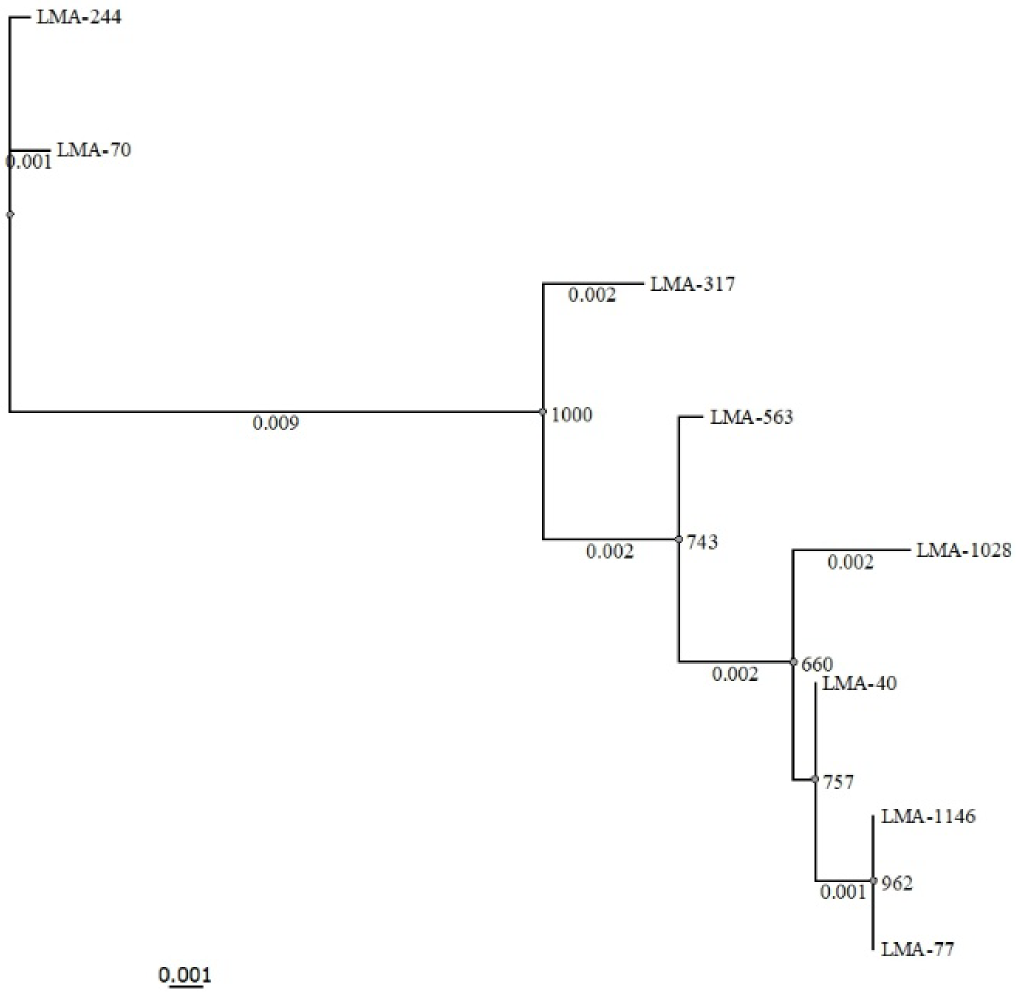


**Figure S2**. Phylograms constructed with the PhyML Maximum-Likelihood method with 1000 bootstraps with 8 isolates of *Geotrichum candidum* for the MLST2013 scheme (*ALA1, CDC19, ERG10, GLN4, PGI1*, *PGM2*). Phylogenetic analyses were conducted in UGENE v1.31.1.

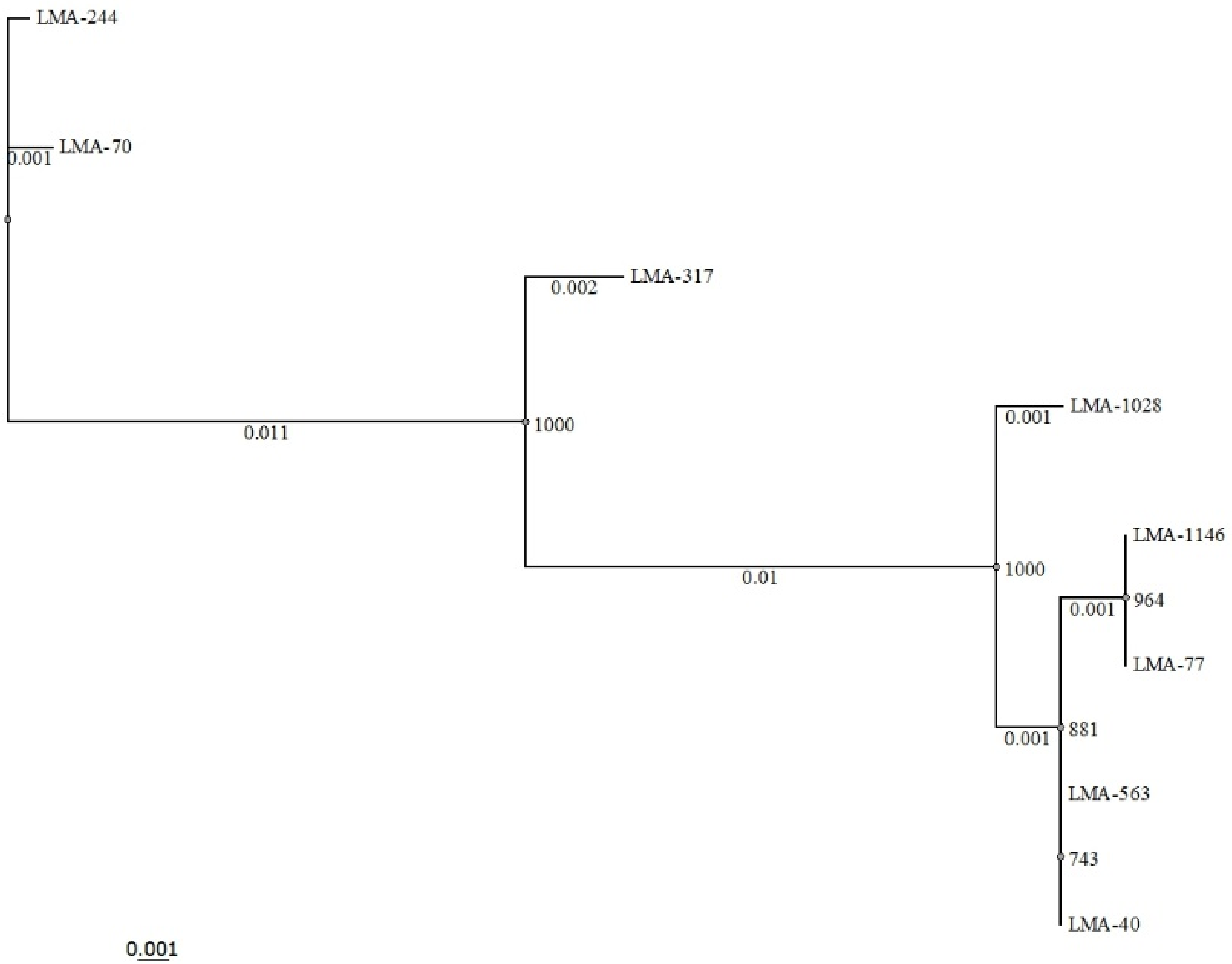


**Figure S3**. Phylograms constructed with the PhyML Maximum-Likelihood method with 1000 bootstraps with 8 isolates of *Geotrichum candidum* for the consensus MLST (MLST2019) developed in this study (*ALA1*, *CDC19*, *SAPT4*, *GLN4*, *PGI1*, *PGM2*). Phylogenetic analyses were conducted in UGENE v1.31.1.

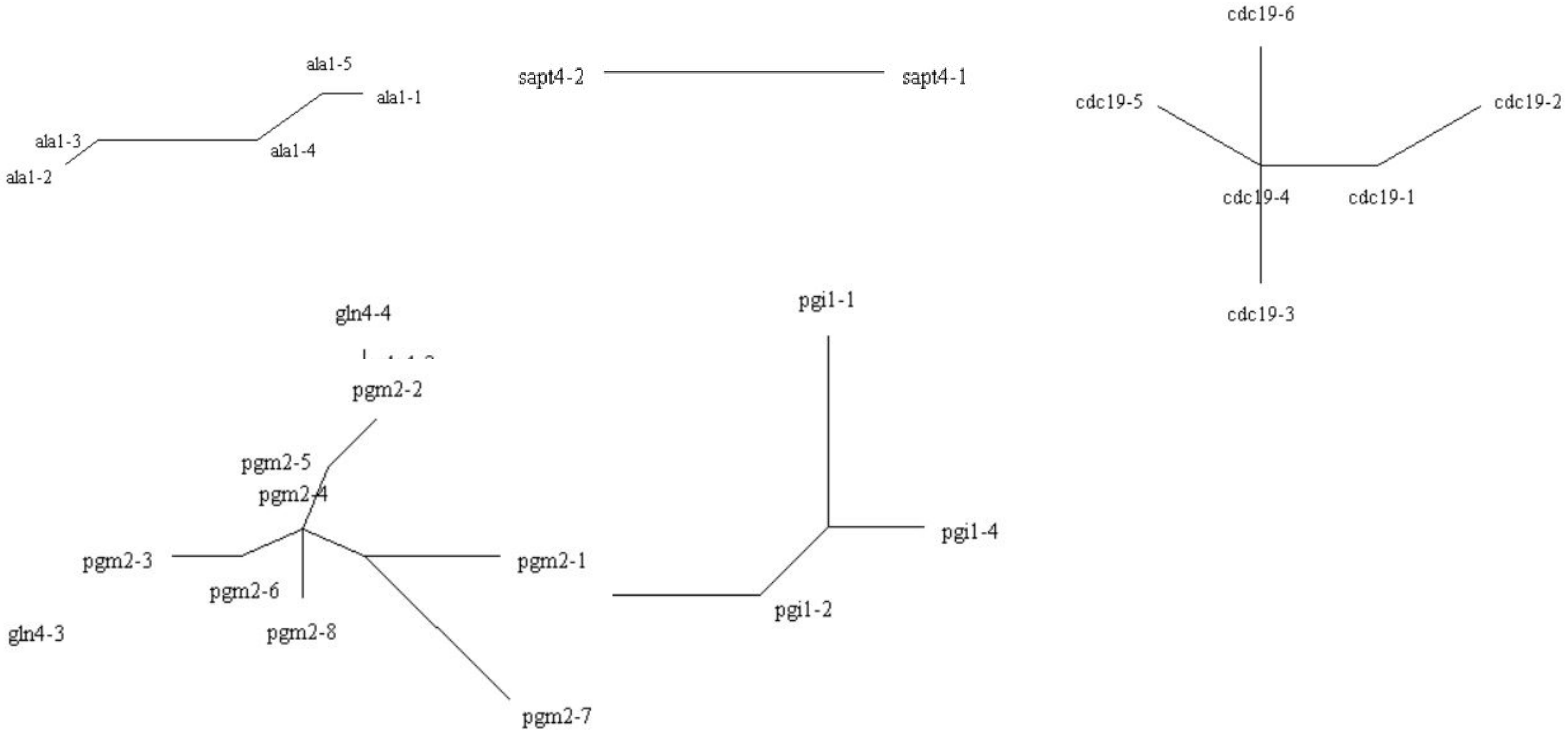


**Supplementary Figure S4**. Split decomposition analysis of the alleles obtained from 41 *Geotrichum candidum* strains from six loci (*ALA1*, *CDC19*, *SAPT4*, *GLN4*, *PGI1*, *PGM2*). Numbering in the figure corresponds to alleles number. Parallelogram formation indicate recombination events. Split decomposition analysis was performed with SplitsTree version 4.
